# Grand Challenges for Public Health Education and Promotion

**DOI:** 10.3389/fpubh.2022.917685

**Published:** 2022-06-27

**Authors:** Christiane Stock

**Affiliations:** ^1^Institute of Health and Nursing Science, Charité–Universitätsmedizin Berlin, Corporate Member of Freie Universität Berlin and Humboldt-Universität zu Berlin, Berlin, Germany; ^2^Unit for Health Promotion Research, University of Southern Denmark, Esbjerg, Denmark

**Keywords:** health education, health promotion, settings based, co-creation, health literacy, digital health (e-health)

## Introduction

The Section Public Health Education and Promotion aims to advance the scientific basis of knowledge and action for current and future health education and promotion researchers and professionals, including those working with lay audiences. The section seeks to foster research on a broad range of health education, health promotion and disease prevention approaches operating at individual, organizational, community and society level. The Ottawa Charter (1) has provided a basis for addressing public health challenges in fundamentally new ways with building public policy, creating supportive environments for health, strengthening community action, developing personal skills and reorienting health services. However since the Charter has been launched many social and economic changes have occurred and globalization as well as digitization have had strong impacts on systems, governance structures, economies and therewith on the daily lives of people. Therefore, the conference on health promotion in 2016 has released the Shanghai Declaration on promoting health in the 2030 Agenda for Sustainable Development (2), which suggests that health promotion methods and strategies play a key role to achieve the UN Sustainable Development Goals (SDGs) (3) and the Declaration puts Healthy Cities, improved governance for health and health literacy in the focus of actions. More recently, the COVID-19 pandemic has reminded us that health is multi-faceted and that there are multiple and interacting impacts of the pandemic in the settings of our everyday lives (4). Therefore, the Section Public Health Education and Promotion aims to foster innovative research, education and practice that can help individuals to better deal with the challenges of rapidly changing environments and make better personal health choices, health education and promotion professionals more effectively engage in evidence-based practices, and societies enhance programmatic efforts and policy initiatives to protect and promote population health. In order to support the goals of the Ottawa Charter and the Shanghai Declaration, the following eight topics and themes should be addressed in future research to create better evidence for tackling the present and future public health challenges effectively.

## Health Promotion in Settings

The importance of settings as “.. a place or context in which people engage in daily activities in which environmental, organizational and personal factors interact to affect health and wellbeing” (5) for sustainable health and wellbeing has been expressed in many different WHO documents since the Ottawa Charter has shed the light on the settings-based approach to health promotion in 1986. The importance of settings is presently re-affirmed, because the recovery from COVID-19 will to a large extent be determined by and experienced in the settings in which we live our lives, and a settings approach is thus needed to mitigate the negative impacts of COVID-19 (6). Whitelaw et al. (7) have distinguished different types of setting based approaches and provided a better understanding of how setting-based health promotion is implemented in current practice. The types range from the passive model, where the setting provides access to population groups or individuals to address health behavior change and development of personal skills, to the organic model requiring active engagement of the community and the comprehensive model, where health promotion is the central component of the setting development. In the settings-based approach to health promotion participation and empowerment of both individuals and communities is key. However, more research is needed to investigate the effectiveness of the different types of health promotion within settings on health and wellbeing of the members of the setting and the wider communities with which settings are deeply interconnected (8).

## Health Literacy With Focus on the Health Literacy Environment

The last two decades have seen an extraordinary increase in published papers on the subject of health literacy showing the growth in interest in this topic. Health literacy has been described as the ability to assess, understand, appraise and apply health-related information in the domains of health promotion, prevention and health care (9). There is a body of research showing that health literacy has influence on behavioral choices and the use of health services, which in turn creates an impact on health outcomes and health service costs (10–12). Given the relevance of health literacy to improve population health there is still a lack of research both in terms of measuring the level of health literacy as well as to improve health literacy in diverse populations. The focus of the majority of studies has been on interventions providing information, effective communications and structured education in clinical settings and directed toward improving functional health literacy, while implementation of national policies and programs still seems to be lacking behind (13). In addition, more research is needed on health literacy environment approaches encompassing programmes, interventions and policies to make health services more user-friendly and to reduce communication barriers.

## Digital Health Education and Promotion

Since technology is shaping nearly every aspect of our lives in information societies health education and health promotion is also transitioning into a new technological and digital era and digital health promotion is evolving (14, 15). While the technology is rapidly developing and improving and the connectivity and adoption of devices is increasing world-wide, there are many new options to reach out to hard-to-access populations and communities in better and more affordable ways. Another advantage is the potential use of new ways to communicate digitally with tailored and even individually personalized health information and messages, health promotion services. With the decreasing costs of digital technologies reaching out to large populations with digital health promotion seems to be possible even in low- and middle income countries. However, successful digital health promotion needs to take some environmental factors into account that are minimizing still existing digital divides in terms of access, the need to develop applications that are easy to use across generations and age groups, to incorporate interactivity and gamification elements, to deliver incentives for use in real time and to establish trust in terms of high standards of data protection (15). A scoping review has shown that digital health promotion uses a variety of technologies ranging from computer- and web-based programs to mobile devices/smartphone apps and telemonitoring in form of sensors (14). However, the authors also conclude that there is still a lack of research of environmental and structural approaches in the field of digital health promotion and that most applications and programs operating with digital technologies focus on individual behavior change (15). In addition, more research is needed to address key challenges of digital health promotion and education in terms of privacy control, appropriate use of data including secondary usage beyond the original intention and the appropriate limits of nudging vs. the free choice (15).

## Co-Creation in Intervention Development and Stakeholder Engagement

It is widely accepted that co-creation of new interventions and stakeholder involvement has the potential to develop more effective interventions with strong and enduring impact on health outcomes (16) and to speed up the application in practice (17). Co-creation has emerged from the participatory design paradigm (18) and ensures that programs are designed with those that are ultimately the recipients of a health intervention (19). While Co-creation has been initially used in developing health care services (20) it may also be a promising strategy to adopt to address other complex health behaviors (19). Interventions and programmes developed in co-creation with stakeholders, users and recipients of programmes have thus demonstrated value for researchers, users and society at large (21). In addition, the need to include wider stakeholder groups during the intervention development process has been identified more recently in order to ensure that user generated ideas are feasible and applicable in practice (22). Co-creation processes ensure that emphasis is placed on empowering participants and that all solutions emerging from co-design are user centered and stakeholder supported. The participation and engagement of stakeholders and/or programme recipients in various stages of the intervention development process, has different origins but shares important assumptions and operating principals (16, 23). While collaboration and engagement with users and stakeholders during intervention development processes are considered vital, clear articulation of procedures and considerations for various co-creation methodologies warrants further research attention (19).

## Social Marketing Approaches

Health education and health promotion build to a large extent on research and approaches developed within social marketing. Co-creation e.g., has its roots in participatory action research, but also in co- design originating from service design thinking in marketing (19). Moreover, social norms approaches are widely used to shift risk-taking behavior toward more responsible health behavior, which have their origin in social marketing. While social marketing approaches and techniques are of high value and relevance for health promotion practitioners and researchers, the sharing of knowledge and practices between these fields of science should be intensified. Thus, more social marketing research should be published in the Section Public Health Education and Promotion in order to make innovative social marketing approaches more visible for public health scientists and practitioners.

## Health Communication and Risk Communication

Significant communication components are involved in the management of public health issues. This became even more urgent during the COVID-19 pandemic. Communication strategies are needed to effectively prompt warnings about risks, increase self-efficacy of individuals for behavioral change, and inform about symptomatology and medical treatment. Risk communication is a science-based approach for communicating effectively in such high concern situations than in a pandemic, and it is based on a multi-level process of interactive exchange of information between public government and citizens (24). More research is needed to fully understand how multiple messages about the nature of the risk and about the legal and the institutional arrangements for risk management can be effectively communicated to broad and diverse audiences in times of fake news and misinformation.

## New Ways to Evaluate Health Education and Promotion Programmes

The last decades have seen an increasing emphasis on evidence-based programmes and actions in the field of health promotion and prevention. However, developing such evidence is still a challenge given the complex nature of many of the community or settings-based interventions with multiple intervention strategies and the diversity of the outcomes on both behavioral and structural level. Since the randomized-controlled trial (RCT) as the gold standard in intervention evaluation is often too restrictive to fit to the diverse types of interventions, the research methods to evaluate their success need to vary according to the type of intervention including qualitative, quantitative, economic and participative methods (25). In addition, new ways to evaluate the effectiveness of digital health tools are needed that allow for capturing dynamic changes of digital health interventions over time (26). Additionally, more research is needed in developing evaluation strategies to tackle the lengthy and costly nature of RCTs.

## Innovative Teaching and Learning in Health Education and Promotion

The multiple and rapid societal end environmental changes in the context of globalization and digitisation require also adaptations and responses in how we teach and learn in the field of health education and promotion. Moreover, the COVID-19 pandemic has changed the way we teach and learn fundamentally with the rapid enforcement of online teaching in many countries. Research addressing the short-, mid- and long-term consequences of distance learning practices on learning outcomes and social interaction among peers and between educators and students are needed. In addition, research about new ways to teach and learn interactively via distance, hybrid or classroom teaching is also warranted.

## Author Contributions

The author confirms being the sole contributor of this work and has approved it for publication.

## Conflict of Interest

The author declares that the research was conducted in the absence of any commercial or financial relationships that could be construed as a potential conflict of interest.

## Publisher's Note

All claims expressed in this article are solely those of the authors and do not necessarily represent those of their affiliated organizations, or those of the publisher, the editors and the reviewers. Any product that may be evaluated in this article, or claim that may be made by its manufacturer, is not guaranteed or endorsed by the publisher.
